# The Ecological Trap: Biodegradable Mulch Film Residue Undermines Soil Fungal Network Stability

**DOI:** 10.3390/microorganisms13092137

**Published:** 2025-09-12

**Authors:** Maolu Wei, Yiping Wang, Feiyu Xie, Qian Sun, Huanhuan Shao, Xiaojie Cheng, Xiaoyan Wang, Xiang Tao, Xinyi He, Bin Yong, Dongyan Liu

**Affiliations:** 1 Key Laboratory of Land Resources Evaluation and Monitoring in Southwest, Sichuan Normal University, Ministry of Education, Chengdu 610101, China; 2 College of Life Sciences, Sichuan Normal University, Chengdu 610041, China

**Keywords:** maize rhizosphere soil, biodegradable mulch film, microplastics, soil fungal network stability, ecological trap, functional replacement

## Abstract

Biodegradable mulching films are promoted as alternatives to traditional polyethylene films, but their environmental impacts remain controversial. This study investigates how biodegradable films affect microplastic pollution of soil, fungal community structure, and ecological network stability. We conducted a maize field experiment comparing conventional polyethylene (CF, PE) and biodegradable (BF, PLA + PBAT) film residues. We used scanning electron microscopy and high-throughput sequencing of fungal ITS genes. We assessed soil properties, microplastic release, fungal communities, and network stability through co-occurrence analysis. BF degraded rapidly, releasing microplastic concentrations much higher than CF. BF increased soil carbon and nitrogen and substantially enhanced maize biomass. However, it significantly reduced soil pH and decreased key functional fungi (saprotrophs and symbionts) abundance. The fungal ecological network complexity and stability declined significantly. Correlation analysis revealed positive associations between saprotrophic and symbiotic fungi abundance and network stability. In contrast, CF reduced some nutrient levels but improved fungal network complexity and stability. This study reveals that biodegradable films create an “ecological trap.” Short-term nutrient benefits mask systematic damage to soil microbial network stability. Our findings challenge the notion that “biodegradable equals environmentally friendly.” Environmental assessments of agricultural materials must extend beyond degradability to include microplastic release, functional microbial responses, and ecological network stability.

## 1. Introduction

Plastic mulch films are widely used in modern agriculture for their outstanding ability to increase soil temperature, conserve moisture, suppress weeds, and boost crop yields [1,2]. As reported, the global utilization of agricultural plastic amounted to 7.4 million tons in 2019 [3]. However, conventional films, such as those made of polyethylene (PE), are not readily biodegradable. Their long-term residue in farmland not only leads to severe environmental pollution [4], but also has a negative effect on crop growth, such as altering root characteristics and reducing crop yields [5,6]. To address this challenge, biodegradable films made from materials like polylactic acid (PLA) and poly (butylene adipate-co-terephthalate) (PBAT) have been promoted as environmentally friendly alternatives [7]. While these films offer the same basic agricultural benefits and are designed to degrade into carbon dioxide and water [8], their actual degradation is often suboptimal under real-world environmental conditions [9]. Consequently, large quantities of partially degraded film fragments break down into microplastics (MPs), particles smaller than 5 mm in diameter through processes like wind-induced mechanical breakdown and dispersal, water runoff-induced fragmentation and UV radiation, causing persistent soil contamination [10,11].

Numerous studies have confirmed the multifaceted impacts of MPs on soil ecosystems. MPs not only alter soil physicochemical properties, such as porosity and pH [12,13], but also influence soil nutrient cycling. Biodegradable MPs have recently been shown to have a dual role in both soil carbon release and stabilization [14]. Additionally, at the microbial level, studies have demonstrated that MPs can influence soil microbial communities by modifying soil physical and chemical properties and they also regulate soil enzyme activities [15,16,17]. Moreover, soil nutrient conditions and plant biomass are affected by MPs [6,15,18]. Previous studies have showed that the diversity and abundance of rhizosphere microbial communities are significantly affected by MP type, dosage, and exposure time [19]. For example, the addition of different-sized PE film residues has been found to decrease the relative abundance of both bacterial and fungal communities in rice rhizosphere soil [4]. In cotton fields, increasing concentrations of PE residues reduced fungal abundance, with a more pronounced effect on rhizosphere fungi [20]. Furthermore, prolonged mulching leads to the accumulation of film residues and MPs [21,22], which can further diminish microbial diversity [23]. However, findings on the microbial impacts of conventional film residues are inconsistent, with reports of positive [24], negative [25,26], and non-significant effects [27]. Similarly, biodegradable film residues can also alter the composition and diversity of microbial communities [28] and change the potential functions of fungal communities [29].

Soil microorganisms, especially fungi are critical components of soil ecosystems and core functional groups for maintaining soil health, nutrient cycling and fertility [30]. Both conventional and biodegradable film residues impact these microbial communities. Yet, most research has focused on bacterial communities [31,32,33], with comparatively fewer studies on fungi. Moreover, previous studies have often relied on pot experiments or simulated field conditions [34,35]. While providing important baseline data, these approaches have left our understanding of the long-term effects of plastic residues on fungal communities in real agricultural settings limited. The impact of plastic film residues can also vary significantly with crop type and tillage systems [13,36,37]. Crucially, existing research has largely overlooked changes in microbial community network structures, particularly the role of key functional groups in maintaining network stability. Therefore, investigating the effects of different film residues on soil properties, fungal communities and network functions is essential for clarifying the mechanisms and risks of plastic film contamination in agroecosystems.

Biodegradable films warrant special attention. Does their faster degradation rate lead to a greater release of MPs compared to conventional films? Could the accumulation of these MPs act as a double-edged sword, creating an ecological trap by providing short-term nutrient benefits while disrupting the ecological network structure and stability through interference with key fungal functional groups? To test this hypothesis, we conducted a field experiment with maize (*Zea mays* L.). The experiment simulated soil residue accumulation after more than 30 years of plastic film mulching. We systematically compared the effects of conventional and biodegradable film residues on soil physicochemical properties and fungal communities. Our study aimed to reveal the in situ impacts and mechanisms of plastic residues on fungal communities and their potential functions. This research aims to provide a new perspective and scientific basis for understanding how soil fungal communities respond to different types of plastic residues and for clarifying their ecological consequences.

## 2. Materials and Methods

### 2.1. Study Site Description

The field experiment was conducted at the Qiushi Farm on the Chenglong Campus of Sichuan Normal University in Chengdu, Sichuan Province, China (30°34′ N, 104°11′ E). This region lies in the subtropical humid monsoon climate zone, marked by a mild climate and clear seasonal changes. The average annual high and low temperatures are 22 °C and 14 °C, respectively, with an annual precipitation of approximately 771.8 mm. The soil is primarily a mixture of loam and clay, with a pH value maintained between 6.5 and 7.0, containing 2–4% organic matter. The accumulated annual temperature ranges from 4600 to 5000 °C, making the region suitable for cultivating a variety of crops [38]. The experimental plot, measuring 40 × 50 m, was previously used as a seedling nursery for the past 10 years.

### 2.2. Experimental Design and Treatments

We employed a split-plot design. The field was divided into six main plots (10 m × 8 m each), separated by 5 m buffer zones to minimize edge effects. Within each main plot, three treatments were randomly assigned [39], CK (Control): No mulch film application, CF (Conventional Film): Addition of conventional polyethylene (PE) film fragments, BF (Biodegradable Film): Addition of biodegradable polylactic acid/poly (butylene adipate-co-terephthalate) (PLA + PBAT) film fragments.

Both film types had a thickness of 0.01 mm. The PE film was purchased from ChengDuJiuZhouFengLe Agricultural Technology Co., Ltd. (Chengdu, China), and the PLA + PBAT film was from Jialemi Gardening Technology Co., Ltd. (Shaoxing, China). The application rate was calculated to simulate the residue accumulation from 32 years of continuous mulching in farmland [40]. Film fragments of two size classes (<4 cm^2^ and 4–25 cm^2^) were manually incorporated into the top 0–30 cm of soil. For the CF treatment, PE fragments were added at 360 kg/ha. For the BF treatment, PLA + PBAT fragments were added at 180 kg/ha [39,41], while the CK treatment underwent the same land tillage treatment without the addition of plastic film fragments. Before film application, a one-time basal fertilizer was applied to all plots at a N:P:K ratio of 280 g:112.7 g:180 g, using a mixture of potassium nitrate and urea as the nitrogen source [42], phosphorus pentoxide as a phosphorus source. Supplemental fertilization was applied as needed based on crop growth requirements [43].

### 2.3. Sample Collection

Maize (cultivar Xingyu90) was sown in April 2024. Sixty days after transplanting (at the tasseling stage, BBCH 50–59), both plant and soil samples were collected. Soil samples were collected from the 0–20 cm depth layer using a five-point mixed sampling method. Following the removal of surface debris, use a soil auger to collect rhizosphere soil from the maize root zone, operationally defined as soil adhering to fine roots. Then pool the five subsamples to form a composite replicate in each plot. A total of 18 samples were collected from six replicate plots encompassing three treatments. Each composite sample was screened through a 2 mm sieve and divided into two subsamples: one was air-dried and ground for physicochemical analysis, and the other was stored at −80 °C for DNA extraction [41].

### 2.4. Analysis of Film Degradation and Microplastic Concentration

Scanning electron microscopy (SEM; SU8010, Hitachi, Tokyo, Japan) was used to observe the morphological characteristics of new CF and BF fragments, CF residues aged in soil for 1.5 years and BF residues aged in soil for 20 days. Microplastics (MPs) were extracted from soil using a density separation method [44]. The concentration of MPs was then determined gravimetrically by weighing the filtered residue.

### 2.5. Measurement of Soil Properties

Plant biomass was quantified by direct fresh weight measurement. Soil water content was determined by oven-drying at 105 °C. Soil pH was measured in a 1:2.5 soil-to-water suspension. Soil bulk density was assessed using the core ring method on a dry weight basis. Total carbon (TC) was quantified by potassium dichromate oxidation, and total nitrogen (TN) was determined by an improved high-temperature digestion and UV spectrophotometric method [45]. Briefly, 0.25 g air-dried soil was moistened with 1 mL water, mixed with 4 mL concentrated H_2_SO_4_, pre-digested overnight and digested at 380 °C until dark. H_2_O_2_ was then added dropwise until clear. After 2 h at room temperature, absorbance at 625 nm was read on a microplate reader (Spectra Max i3x, Molecular Devices, Shanghai, China). Inorganic N was extracted from 5 g fresh soil with 25 mL 2 M KCl by shaking for 1 h and filtering. NO_3_^−^-N was quantified by dual-wavelength UV (220/275 nm). NH_4_^+^-N was measured by the indophenol blue colorimetric method at 650 nm [46]. Dissolved organic carbon (DOC) and nitrogen (DON) were extracted with a 0.5 M K_2_SO_4_ solution (1:5 *w*/*v*), and quantified using the same methods applied for TC and TN.

### 2.6. DNA Extraction and Fungal ITS High-Throughput Sequencing

Total genomic DNA was extracted from 0.5 g of each soil sample following the method described by [47]. The integrity and concentration of the extracted DNA were verified using 0.7% agarose gel electrophoresis and a NanoDrop 2000 spectrophotometer (Thermo Fisher Scientific, Waltham, MA, USA), respectively. The ITS2 region of the fungal ribosomal DNA was amplified using the specific primers ITS3: GATGAAGAACGYAGYRAA and ITS4: TCCTCCGCTTATTGATATGC [48]. The purified amplicons were sequenced on an Illumina PE250 platform.

### 2.7. Bioinformatic and Statistical Analysis

Raw sequencing reads were merged using FLASH and demultiplexed. Quality control was performed with QIIME2, filtering out sequences with a mean quality score < 30, length < 200 bp, or containing ambiguous bases (N). Sequence denoising and chimera removal were conducted using the DaDa2 algorithm in QIIME2, generating an Amplicon Sequence Variant (ASV) feature table. Taxonomic assignment was performed against the SILVA database (v138) using a Naïve Bayes classifier. A phylogenetic tree was generated using FastTree within QIIME2. To normalize sequencing depth, all samples were rarefied to the minimum number of sequences per sample. Raw reads were deposited in the NCBI Sequence Read Archive database (PRJNA1307480).

Statistical analyses were performed in R (v4.3.3). Alpha diversity indices were calculated with the vegan package. The Wilcoxon rank-sum test (wilcox.test in the stats package) was used for two-group comparisons, while the Kruskal–Wallis test followed by post hoc multiple comparisons (agricolae package) was used for multi-group comparisons. Asterisks indicate significance in all figures: * *p* < 0.05, ** *p* < 0.01, *** *p* < 0.001, **** *p* < 0.0001, ns indicates no significant difference. Beta diversity was evaluated using Bray–Curtis distances (vegdist in vegan) and visualized with Principal Coordinates Analysis (PCoA) (vegan packages). Permutational multivariate analysis of variance (PERMANOVA) was conducted using the adonis function (vegan). Fungal functional guilds were predicted using FUNGuild.

### 2.8. Co-Occurrence Network Analysis

Soils represent highly complex ecosystems where microbial communities play critical roles in nutrient cycling, plant health, and environmental resilience. To unravel the intricate interactions within these communities, co-occurrence network analysis has emerged as a powerful tool [49,50]. Co-occurrence networks were constructed for each treatment based on their respective ASV tables. A connection (edge) was established between two nodes (ASVs) if Spearman’s rank correlation coefficient was >0.8 or <−0.8 with a Bonferroni-corrected *p*-value < 0.05. Network complexity was evaluated using topological parameters including total nodes, edges, network diameter, relative modularity, average clustering coefficient, and average degree, computed using the igraph package. Visualization of the networks was performed with Gephi (v0.10.1). We assessed the potential network stability by using multiple metrics, including simulating random (50% of nodes) and targeted nodes removal to measure robustness. And the cohesion of each network was calculated based on the proportion of positive correlations using a null model approach. Network stability was further quantified as the ratio of absolute negative cohesion to positive cohesion [51].

## 3. Results

### 3.1. SEM Analysis of Film Change and Soil Microplastic Concentration

**Figure 1:**
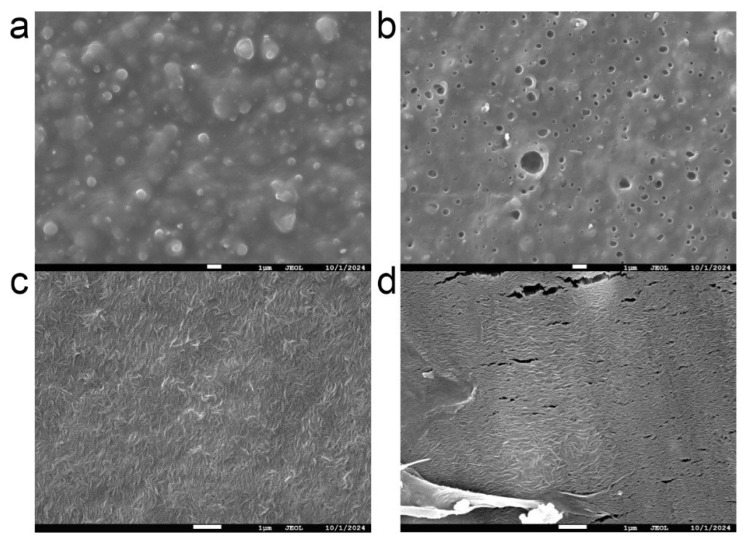
Comparison of SEM images of conventional and biodegradable mulch films in soil. (**a**) New CF film, (**b**) CF film after 1.5 years in soil, (**c**) New BF film, (**d**) BF film after 20 days in soil. CK (no mulch film application), CF (addition of PE film fragments); BF (addition of PLA + PBAT film fragments).

To visualize the differential degradation of the two mulch films in soil, we observed their surface morphology using scanning electron microscopy (SEM) (Figure 1). The SEM images clearly revealed disparate degradation rates between the CF and BF treatments. Before soil application, the surfaces of both new CF (Figure 1a) and new BF (Figure 1c) films were relatively smooth and intact, with no obvious pores or cracks. After 1.5 years of incubation in the soil, the surface of the CF exhibited numerous pores and cracks. However, its macroscopic structure remained largely intact (Figure 1b) indicating strong durability and high physical inertia. This suggests that CF primarily acts as a slow-releasing source of microplastics and a persistent physical barrier. In contrast, the BF film showed extensive cracking and fragmentation after only 20 days in the soil (Figure 1d). Its original surface structure had almost completely disintegrated. This indicates that the degradation of the BF is accompanied by the rapid release of its components (e.g., polylactic acid, plasticizers) and microplastics, introducing a substantial amount of readily degradable organic matter and small particles into the soil environment in a short period.

**Figure 2:**
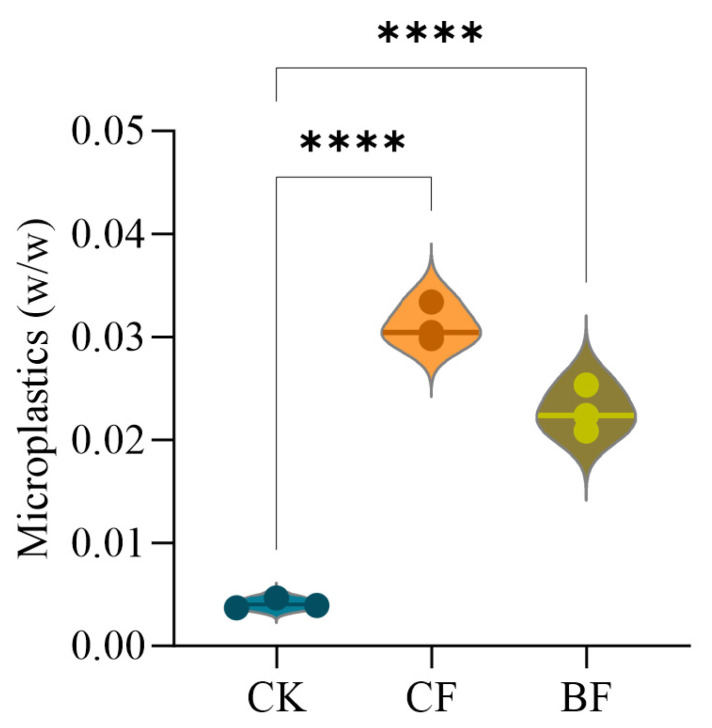
Microplastic concentration in soil. Values are represented as the mean ± standard error (*n* = 3). CK (no mulch film application), CF (addition of PE film fragments); BF (addition of PLA + PBAT film fragments). **** *p* < 0.0001.

To quantify the intensity of microplastic input corresponding to these different degradation patterns, we measured the microplastic concentrations in the soil of each treatment. The findings indicated that microplastic concentrations were notably higher in both the CF and BF treatments compared to the CK (*p* < 0.0001, Figure 2). The microplastic concentration in the BF treatment reached approximately 0.031%, which was notably higher than the concentration in the CF treatment (approximately 0.023%). This evidence demonstrates that the rapid degradation of the BF did not lead to complete mineralization. Instead, it led to an accelerated fragmentation rate leading to a greater input of secondary microplastic particles into the environment.

### 3.2. Differences in Soil Microenvironment Induced by Film Residues

The different types of film residues had significantly different impacts on the physicochemical properties of the soil (Table 1). Under the CF treatment, soil acidity increased, with the pH decreasing from 6.31 to 6.14 (*p* < 0.05). DON decreased from 0.20 to 0.13 g/kg (*p* < 0.05), indicating reduced nitrogen turnover activity. Although SWC, NH_4_^+^-N, NO_3_^−^-N and TN all showed a decreasing trend under the CF treatment, these changes were not statistically significant (*p* > 0.05). TC and DOC increased slightly, but not significantly (*p* > 0.05). In contrast, BF treatment had a more pronounced effect on the soil microenvironment. The soil pH dropped sharply from 6.31 to 5.79 (*p* < 0.05), highlighting a strong acidification effect. The TC content increased from 45.89 to 51.36 g/kg (*p* < 0.05) and NO_3_^−^-N increased from 12.86 to 14.27 mg/kg (*p* < 0.05). The concentrations of NH_4_^+^-N, DON, and DOC were also higher than in CK, though these increases did not reach statistical significance (*p* > 0.05). Meanwhile, SWC and TN levels remained comparable to the CK. Overall, the CF residue primarily resulted in mild soil acidification and decreased nitrogen activity, with limited impact on the main soil carbon and nitrogen pools. The BF residue, however, led to the rapid accumulation of carbon and available nitrogen, accompanied by significant acidification.

### 3.3. Response of Rhizosphere Fungal Community to Film Residues

**Figure 3:**
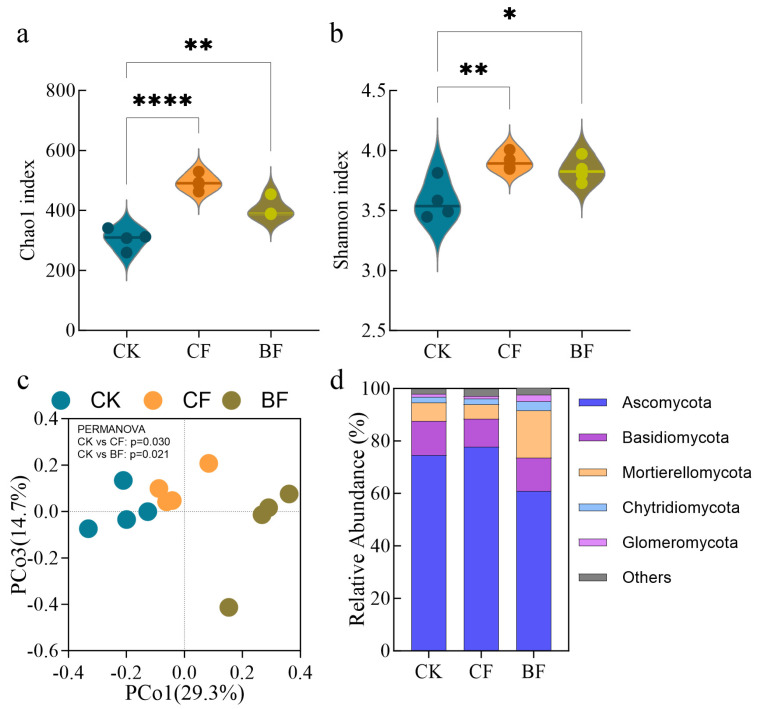
Rhizosphere soil fungal community analysis. (**a**) Chao1 index, (**b**) Shannon index at the ASV level, (**c**) Principal Coordinates Analysis (PCoA) and (**d**) relative abundance at the phylum level. Values are represented as the mean ± standard error (*n* = 4). CK (no mulch film application), CF (addition of PE film fragments); BF (addition of PLA + PBAT film fragments). * *p* < 0.05, ** *p* < 0.01 and **** *p* < 0.0001.

A comparison of fungal community diversity and composition revealed that the addition of both film types significantly increased alpha diversity parameters (Figure 3a,b). Compared to the CK, the CF treatment significantly increased the Chao1 richness index (61.6%, *p* < 0.0001) and the Shannon diversity index (32.7%, *p* < 0.01). Similarly, the BF treatment also led to significant increases in the Chao1 index (9.1%, *p* < 0.01) and the Shannon index (7.1%, *p* < 0.05). These findings suggest that film residues, particularly from conventional PE film, positively regulated the species richness and diversity of the soil fungal community.

PCoA based on Bray–Curtis distances further revealed that each type of film residue drove distinct fungal community succession (Figure 3c). The PCo1 and PCo3 axes explained 29.3% and 14.7% of the total variation, respectively, accounting for a combined 44.0% and indicating that the treatments significantly drove the differentiation of fungal community structure. The PCoA plot shows that samples from the CK, CF and BF treatments formed clearly separated clusters. This separation was statistically confirmed by PERMANOVA, which showed that the community compositions of both CF and BF were significantly different from CK, reflecting that film application substantially altered the overall structure of the rhizosphere fungal community.

To identify the key taxa driving this community differentiation, we conducted a taxonomic analysis at the phylum level (Figure 3d). Ascomycota was the dominant phylum across all samples, followed by *Basidiomycota*, *Mortierellomycota*, *Chytridiomycota* and *Glomeromycota*, with these phyla collectively accounting for 97.5% of the total abundance. Compared to CK, the CF treatment showed a slight increase in the relative abundance of Ascomycota (4.3%), while the relative abundances of *Basidiomycota*, *Mortierellomycota* and *Glomeromycota* decreased by 18.3%, 21.4% and 12.2%, respectively. Though these changes were not statistically significant under CF treatment. In contrast, the BF treatment triggered a dramatic shift in the dominant phyla. The relative abundance of Ascomycota decreased significantly by 18.3% compared to CK. The ecological niche vacated by Ascomycota was subsequently occupied by other phyla, most notably *Mortierellomycota*, Chytridiomycota and *Glomeromycota*, which exhibited explosive increases in abundance of 155.8%, 70.2% and 108.5%, respectively.

**Figure A1:**
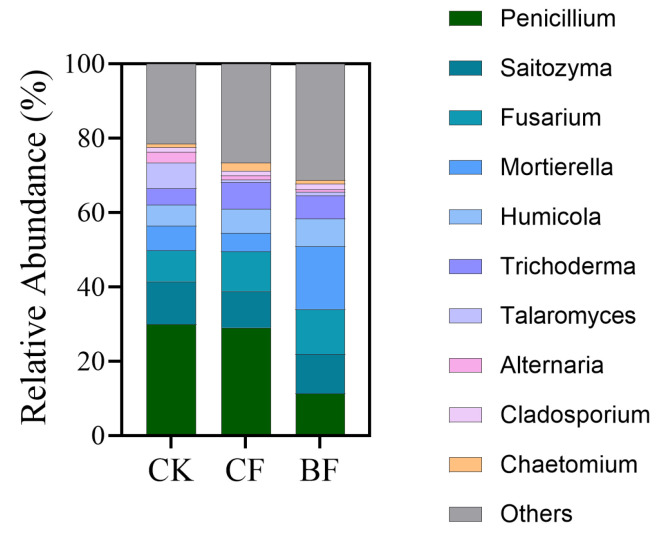
Relative abundance of rhizosphere soil fungal genera in three treatments. CK (no mulch film application), CF (addition of PE film fragments); BF (addition of PLA + PBAT film fragments).

Additionally, the top 10 major genera were analyzed to further characterize community shifts (Figure A1). *Penicillium* was the most dominant genus across all treatments, followed by *Mortierella*, *Saitozyma*, *Fusarium*, *Humicola* and *Trichoderma*. Under the CF treatment, the composition of dominant genera was broadly comparable to CK. While *Fusarium* and *Trichoderma* increased markedly by 27.5% and 61.6%%, respectively. In the BF treatment, the relative abundance of *Penicillium* declined significantly compared with CK, while *Mortierella* increased sharply by 157.0% and emerged as a dominant genus. *Trichoderma* also increased by 36.5%. These changes highlight a pronounced enrichment of beneficial and saprotrophic taxa under BF.

### 3.4. Differentiated Response of Fungal Functional Guilds in Maize Rhizosphere Soil

**Figure 4:**
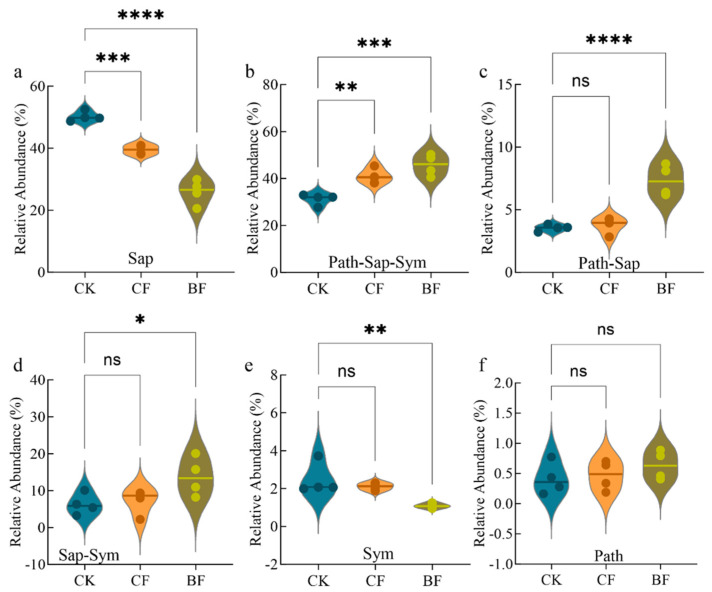
Relative abundance of major fungal functional guilds in the rhizosphere soil. The guilds are abbreviated as (**a**) Saprotroph (Sap), (**b**) Pathotroph-Saprotroph-Symbiotroph (Path-Sap-Sym), (**c**) Pathotroph-Saprotroph (Path-Sap), (**d**) Saprotroph-Symbiotroph (Sap-Sym), (**e**) Symbiotroph (Sym) and (**f**) Pathotroph (Path). Values are represented as the mean ± standard error (*n* = 4). CK (no mulch film application), CF (addition of PE film fragments); BF (addition of PLA + PBAT film fragments). * *p* < 0.05, ** *p* < 0.01, *** *p* < 0.001 and **** *p* < 0.0001, ns indicates no significant difference.

We used FUNGuild to predict the functional roles of the fungal communities and found that the different film residue types had distinct impacts on various functional guilds (Figure 4). Compared to CK, CF treatment had a relatively moderate effect on the main functional groups. The most notable changes were a significant 21.3% decrease in the relative abundance of Saprotroph (Sap) (*p* < 0.001) and a significant 31.8% increase in the multi-functional Pathotroph-Saprotroph-Symbiotroph (Path-Sap-Sym) guild (*p* < 0.01). Other guilds, including Pathotroph-Saprotroph (Path-Sap), Saprotroph-Symbiotroph (Sap-Sym) and Symbiotroph (Sym), showed no significant changes (*p* > 0.05). In contrast, BF treatment induced a much more drastic and systematic restructuring of the fungal functional guilds when compared to the CK. The relative abundance of Saprotrophs decreased dramatically by 48.4% (*p* < 0.0001), indicating strong suppression of this key decomposer community. The abundance of Sym also fell sharply by 55.9% (*p* < 0.01), further weakening the stabilizing role of this keystone functional group. Conversely, the BF treatment led to a significant increase in several multi-functional guilds. The Path-Saprotroph-Sym guild increased by 46.5% (*p* < 0.001). Furthermore, the mixed-guilds of Path-Sap and Sap-Sym showed substantial increases of 106.8% (*p* < 0.0001) and 119.1% (*p* < 0.05), respectively. The abundance of the pure Pathotroph guild did not differ significantly among the three treatments (*p* > 0.05). These findings suggest that the impact of the CF treatment was mainly characterized by a limited redistribution of fungal functional guilds. The BF treatment, however, drove a profound replacement and reconstruction of the entire functional structure of the rhizosphere fungal community.

### 3.5. Ecological Reshaping of Fungal Co-Occurrence Networks by Different Film Residues

**Figure 5:**
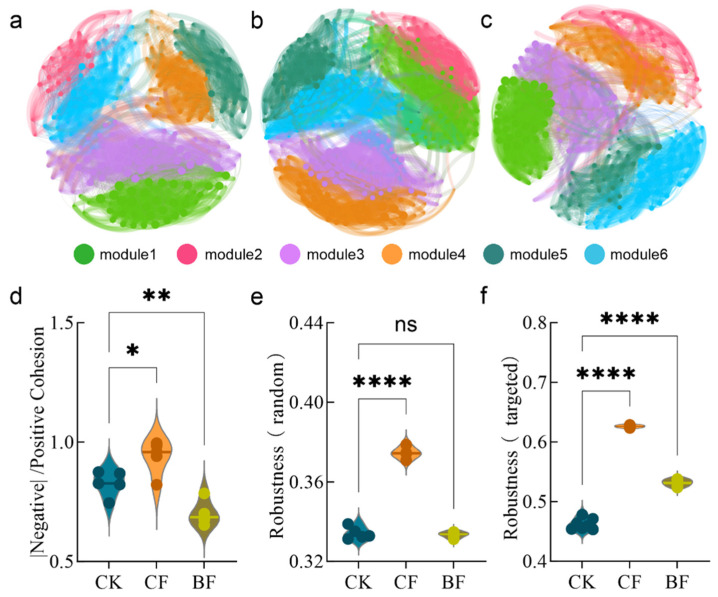
Co-occurrence networks and topological properties of fungal communities in the soil under CK (**a**), CF (**b**), and BF (**c**) treatments. Stability indicators for the soil microbial networks under different treatments, including the absolute ratio of negative to positive cohesion (**d**), robustness under random removal (**e**) and robustness under targeted removal (**f**). CK (no mulch film application), CF (addition of PE film fragments); BF (addition of PLA + PBAT film fragments). * *p* < 0.05, ** *p* < 0.01 and **** *p* < 0.0001, ns indicates no significant difference.

To systematically evaluate the effects of different film residues on the ecological networks of rhizosphere fungi, we generated co-occurrence networks for the CK, CF and BF treatments and analyzed their topological properties (Figure 5, Table 2). Compared to CK, CF residue significantly enhanced the complexity and stability of the fungal network. Topological analysis (Table 2) revealed that under the CF treatment, the number of nodes and edges increased from 236 and 2968 in the CK to 324 and 4960, respectively. The average degree also rose from 25.15 to 30.62. These results indicate that the CF treatment fostered a more tightly knit and complex network of interspecies interactions within the fungal community. Furthermore, the CF network demonstrated superior stability. The network cohesion (complexity), measured as the absolute value of negative to positive cohesion was higher in the CF treatment (Figure 5d, *p* < 0.05). This suggests increased competition and niche differentiation, which may contribute to the overall stability of the network structure. Crucially, the network robustness analysis showed that the CF network was significantly more robust than the CK network under both random and targeted node removal scenarios (Figure 5e,f; *p* < 0.0001), indicating a greater ability to resist external disturbances and maintain structural integrity.

In contrast, BF residue had a detrimental effect on the fungal network. Although the number of nodes in the BF network (254) was slightly higher than in the CK (236), its number of edges (2808) and several other key topological parameters were lower (Table 1). Specifically, the average degree decreased from 25.15 to 22.11, network density dropped from 0.11 to 0.087, and the clustering coefficient fell from 0.29 to 0.22. This suggests that the BF treatment led to a reduction in network connections, resulting in a sparser and more loosely organized structure. Concurrently, the absolute value of negative-to-positive cohesion ratio in the BF network was significantly lower (Figure 5d, *p* < 0.01), indicating weakened competitive interactions within the community. In summary, the CF and BF residues exhibited fundamentally different regulatory effects on the soil fungal network. CF enhanced network complexity, connectivity, and stability, thereby strengthening the ecological functioning of the fungal community. Conversely, BF weakened interspecies connections, leading to network simplification and the potential degradation of ecosystem functions.

### 3.6. Correlation Analysis of Fungal Functional Guilds and Network Stability

**Figure 6:**
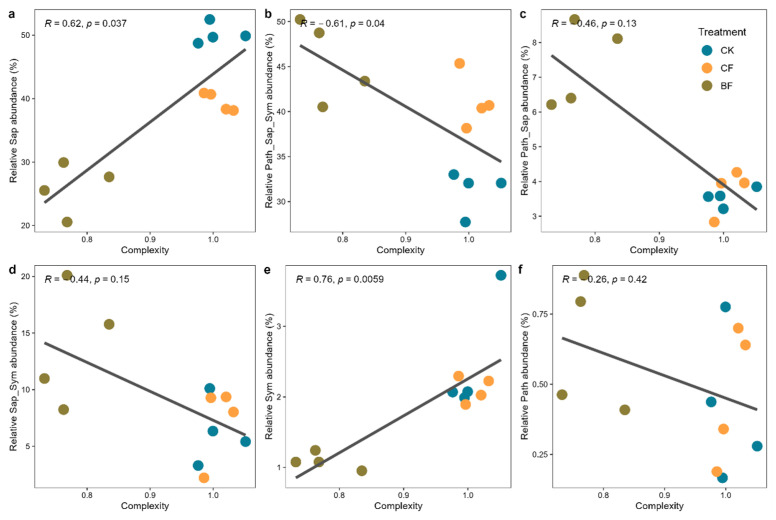
The relationship between fungal functional guilds and network stability. Network stability is assessed by its complexity, defined as the absolute value of negative-to-positive cohesion ratio. The guilds are abbreviated as (**a**) Saprotroph (Sap), (**b**) Pathotroph-Saprotroph-Symbiotroph (Path-Sap-Sym), (**c**) Pathotroph-Saprotroph (Path-Sap), (**d**) Saprotroph-Symbiotroph (Sap-Sym), (**e**) Symbiotroph (Sym) and (**f**) Pathotroph (Path). Values are represented as the mean ± standard error (*n* = 4). CK (no mulch film application), CF (addition of PE film fragments); BF (addition of PLA + PBAT film fragments).

To further elucidate the mechanisms by which different fungal functional guilds regulate the stability of the microbial community network, we conducted a correlation analysis between the relative abundance of each guild and the complexity of the fungal network (Figure 6). The results revealed a significant positive correlation between the Sap guild and network complexity parameters (R = 0.62, *p* = 0.037, Figure 6a), indicating that a higher proportion of Sap is associated with a more complex and robust fungal co-occurrence network. The Sym guild also showed a strong positive correlation with network complexity (R = 0.76, *p* = 0.0059, Figure 6e), indicating that Symplay a key role in enhancing the complexity and stability of the fungal network. Conversely, the multi-functional Path-Sap-Sym guild was significantly negatively correlated with network complexity (R = −0.61, *p* = 0.040, Figure 6b). This suggests that the expansion of this particular guild may weaken the network’s complexity and overall stability. Other mixed-guilds and the parasitic guild including Path-Sap, Sap-Sym and Path also showed negative correlation trends with complexity (Figure 6c,d,f), although these were not statistically significant (*p* > 0.05). In summary, specific core functional guilds (Sap and Sym) contribute to improving the complexity and stability of the fungal network. In contrast, a substantial increase in multi-functional guilds may accelerate network deconstruction, thereby reducing the resilience and sustainability of the soil micro-ecosystem.

### 3.7. Differences in Plant Growth Induced by Film Residues

**Figure 7:**
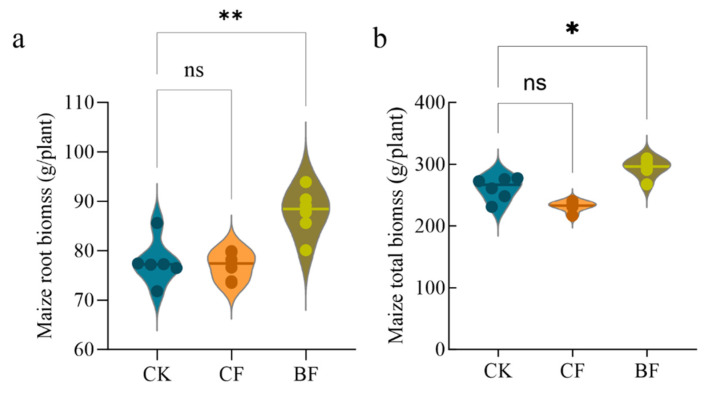
Effects of biodegradable and conventional polyethylene film residues on maize biomass. (**a**) Maize root biomass and (**b**) maize total biomass. Values are presented as the mean ± standard error (*n* = 6). CK (no mulch film application), CF (addition of PE film fragments); BF (addition of PLA + PBAT film fragments). * *p* < 0.05 and ** *p* < 0.01, ns indicates no significant difference.

**Figure A2:**
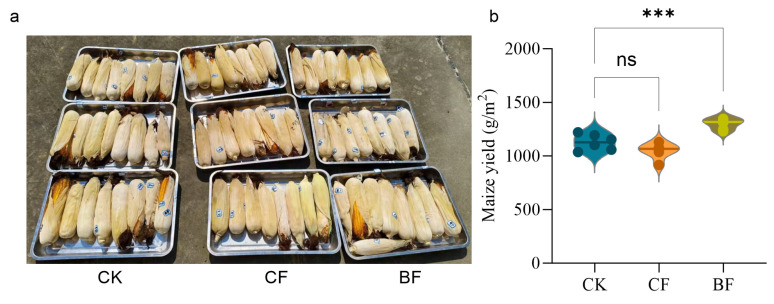
Maize yield in three treatments. (**a**) maize ears, (**b**) maize yield. Values are represented as the mean ± standard error (*n* = 6). CK (no mulch film application), CF (addition of PE film fragments); BF (addition of PLA + PBAT film fragments). *** *p* < 0.001, ns indicates no significant difference.

To investigate the effects of different types of film residues on crop growth, the root and total biomass of maize were measured at 60 days (Figure 7). The results showed that in BF treatment, maize root biomass increased by 13.1%, which was significantly higher than the CK (Figure 7a, *p* < 0.01). The total biomass also showed a significant increase of 12.7% (Figure 7b, *p* < 0.05). This indicates that the BF treatment significantly promoted the growth and matter accumulation of maize. In contrast, the CF treatment showed a non-significant decrease of 0.9% in root biomass and 11.1% in total biomass compared to the CK (*p* > 0.05 for both). This suggests that the direct impact of conventional film residue on plant growth was relatively weak. To evaluate treatment effects on productivity, maize yield was measured under the three treatments (Figure A2). Relative to CK, CF showed a modest, non-significant decrease of approximately 7.0% (*p* > 0.05). By contrast, BF significantly increased maize yield by about 15.3% (*p* < 0.001), indicating a clear yield benefit of the BF treatment.

## 4. Discussion

### 4.1. Biodegradable Mulch as a Significant Source of Soil Microplastic Pollution

SEM images revealed that both CF and BF underwent varying degrees of degradation in the soil (Figure 1). Notably, the BF showed distinct cracks and fragmentation after only 20 days (Figure 1d). Previous research has established that ultraviolet radiation and physical disturbances accelerate the surface cracking and weathering of residual films, thereby promoting the formation of MPs [10,52]. Our findings confirm that the degradation rates of different mulch films vary significantly. The BF residue degraded more rapidly, exhibited a rougher surface, and incurred greater mass loss [53,54]. This directly led to a rapid increase in MP concentration in the short term (Figure 2). This observation is highly consistent with reports suggesting that BF acts as a double-edged sword in the soil, capable of releasing a greater quantity of MPs [14,55,56]. The detection of MPs even in the CK highlights the pervasive nature of their migration and background pollution in agricultural soils [57]. Therefore, the primary finding of this study is that, within the field soil environment, BF paradoxically became a more significant source of MP release than CF.

### 4.2. Impacts of Biodegradable Mulch on Soil Fungal Community Structure and Functional Guilds

The changes in microplastic concentration and the chemical environment, driven by the different film types, inevitably exerted selective pressure on the soil microbial community. The alpha-diversity and PCoA results (Figure 3) demonstrated a strong response from the fungal community. Both CF and BF treatments significantly increased fungal diversity compared to the CK, suggesting that the introduction of mulch films created new ecological niches [58]. The BF, composed of PBAT/PLA material, is known to enrich for fungi involved in the degradation of plastics, such as those from the *Pleosporaceae* family [29].

However, the mechanisms underlying the deeper structural differences in the community are complex. Residual films are known to significantly affect soil pH and physical structure [59,60]. Our study suggests that the pronounced acidification unique to the BF treatment likely resulted from the large quantity of acidic monomers and oligomers produced during its degradation [61], which challenges the physiological limits of many dominant functional groups. The plastic films and their degradation products also provide novel carbon sources for soil microorganisms [62,63,64], with the type of carbon and its accessibility varying with the mulch material, thereby driving a directional selection of the microbial community. Furthermore, the potential toxic effects of additives within the films cannot be overlooked [65,66].

This combination of pressures ultimately led to a “functional replacement” phenomenon within the fungal community (Figure 4). Under the BF treatment, the relative abundance of the key decomposer guild, Sap (primarily Ascomycota), actually decreased. This may be related to an inhibitory effect associated with the unique polymer structure of the BF and its degradation by products [67]. In stark contrast, opportunistic Sap-Sym, such as *Mortierellomycota*, expanded significantly. This could be due to their greater efficiency in utilizing small-molecule organic compounds like lactic acid [54], which aligns with the known tendency of such fungi to colonize the surfaces of degradable films [53].

### 4.3. Decreased Stability and Complexity of the Fungal Ecological Network

The shifting abundances of different functional guilds directly led to the reshaping of the entire fungal ecological network’s structure and stability. Network analysis showed that the complexity and stability of the fungal network were enhanced under the CF treatment, whereas the BF treatment significantly weakened the network structure (Figure 5). Our research and correlation analysis further confirmed that Sap and Sym are core structural groups, with their abundance being highly positively correlated with network complexity and robustness (R = 0.62–0.76, *p* < 0.05) (Figure 6a,e). This finding illustrates that the loss of key functional groups, particularly core decomposers and mutualists, directly undermines network stability.

The ecological mechanisms driving this network differentiation are closely linked to the type of carbon source provided by the mulch films. Conventional PE films primarily release high-threshold additives, which are difficult to metabolize, whereas degradable films release low-threshold carbon sources (such as small-molecule organic compounds) during their breakdown [68]. The BF treatment fostered positive interactions and enhanced cooperation among fungi, reflecting the competitive advantage of microbial collaboration in a resource-rich environment [69]. This also supports the importance of multi-enzyme collaboration in the degradation process of biodegradable films [70,71]. However, both empirical evidence and ecological theory suggest that networks dominated by positive interactions are less stable than those dominated by competition [49]. The competition-dominated structure of the CF network was more stable. In contrast, the cooperation-enhanced BF network was more fragile, exhibiting a significantly lower resistance to external shocks such as species loss or environmental fluctuations (Figure 5) [72].

### 4.4. An Ecological Trap: The Contradiction Between Short-Term Yield Increase and Long-Term Soil Health Risks

Despite its clear negative impacts on the soil fungal network and functional community, the BF treatment unexpectedly resulted in significant increases in soil nutrients and maize growth (Table 1, Figure 7). This nutrient bonus effect likely stems from the rapid input of labile organic compounds and available carbon from BF degradation products [50,73]. Furthermore, these products may have activated relevant enzyme systems, thereby promoting short-term crop growth [18]. This situation creates what can be described as an ecological trap. The system exhibits high nutrient levels and increased yields in the short term, but this comes at the cost of destroying the stability of the microbial network [74]. The coexistence of functional group loss, decreased network complexity and short-term yield gains reflects a gradual erosion of the key soil microbial group [75]. Such network degradation could severely compromise the resilience and long-term productivity of the soil ecosystem when faced with disturbances like drought or disease [76,77].

## 5. Conclusions

This field study systematically reveals the profound mechanisms by which BF reshapes the soil fungal community and ecosystem functions, presenting a strong challenge to the common perception that biodegradable is synonymous with environmentally friendly. Our research discovered that while BF promoted a short-term increase in maize yield, it also significantly elevated the level of microplastic pollution in the soil to 1.3 times that of conventional film. Through the dual physicochemical impacts of microplastic release and the introduction of degradation byproducts, BF drove drastic changes in the soil’s physicochemical environment and microbial community structure. Under the CF treatment, *Fusarium* and *Trichoderma* were more strongly enriched. In contrast, the BF treatment induced a functional replacement within the fungal community. The core saprotrophic and symbiotic functional guilds were suppressed, while opportunistic fungi like *Mortierella* expanded significantly and *Trichoderma* also increased. This shift led to a substantial decrease in the complexity and stability of the soil’s ecological network. Correlation analysis revealed that the loss of these core functional groups was highly associated with the decline in network stability, indicating an erosion of microbial social capital and exposing the ecosystem to a risk of sudden functional collapse. We characterize this phenomenon as an ecological trap, where the short-term nutrient bonus provided by the BF conceals the potential long-term damage to the microbial network and overall soil health. In the long run, the resilience and sustainable functioning of the ecosystem are severely compromised. This study provides an empirical basis and a theoretical framework for the scientific assessment of the environmental safety of degradable agricultural films. This study reflects a single maize-growing season. Future work will include multi-year field trials to assess long-term effects of biodegradable mulch residues on soil functions and investigations into the chemistry of microplastics and degradation byproducts and their impacts on soil microbial communities.

## Figures and Tables

**Table 1:** Physicochemical properties of maize rhizosphere soil.

Parameter	CK	CF	BF
pH	6.31 ± 0.06 a	6.14 ± 0.03 b	5.79 ± 0.15 c
SWC (%)	20% ± 0.02 a	19% ± 0.02 a	19% ± 0.04 a
NH_4_^+^-N (mg/kg)	6.05 ± 1.27 ab	4.53 ± 0.86 b	6.77 ± 0.51 a
NO_3_^−^-N (mg/kg)	12.86 ± 1.87 b	11.01 ± 1.43 b	14.27 ± 1.54 a
DON (g/kg)	0.20 ± 0.03 a	0.13 ± 0.05 b	0.23 ± 0.02 a
DOC (g/kg)	7.83 ± 0.93 a	8.58 ± 0.93 a	8.20 ± 2.2 a
TC (g/kg)	45.89 ± 2.21 b	47.08 ± 1.76 b	51.36 ± 1.17 a
TN (g/kg)	2.68 ± 0.4 a	2.63 ± 0.23 a	2.63 ± 0.2 a

The soil parameters are abbreviated as soil water content (SWC), ammonium (NH_4_^+^-N), nitrate (NO_3_^−^-N), dissolved organic nitrogen (DON), dissolved organic carbon (DOC), total carbon (TC), total nitrogen (TN). Values are presented as the mean ± standard error (n = 4). Different letters indicate a significant difference at *p* < 0.05. CK (no mulch film application), CF (addition of PE film fragments); BF (addition of PLA + PBAT film fragments). Different letters denote statistically significant differences at the *p* < 0.05 level.

**Table 2:** Topological properties of network structure in different treatments. CK (no mulch film application), CF (addition of PE film fragments); BF (addition of PLA + PBAT film fragments).

Network Indexes	CK	CF	BF
Nodes	236	324	254
Edges	2968	4960	2808
Degree	25.15	30.62	22.11
Path length	0.07	0.06	0.07
Diameter	0.15	0.12	0.14
Density	0.11	0.095	0.087
Clustering coefficient	0.29	0.25	0.22
Modularity	0.65	0.66	0.67

## Data Availability

The original contributions presented in this study are included in the article. Further inquiries can be directed to the corresponding authors.
